# Identification of breast cancer-associated PIK3CA H1047R mutation in blood circulation using an asymmetric PCR assay

**DOI:** 10.1371/journal.pone.0309209

**Published:** 2024-08-28

**Authors:** Dinh Thi Thao, Nguyen Phu Thanh, Dong Van Quyen, Ly Tuan Khai, Le Huu Song, Ngo Tat Trung

**Affiliations:** 1 Center for Genetic Consultation and Cancer Screening, 108 Military Center Hospital, Hai Ba Trung, Hanoi, Vietnam; 2 University of Science and Technology of Hanoi, Cau Giay, Hanoi, Vietnam; 3 Institute of Biotechnology, Vietnam Academy of Science and Technology, Cau Giay, Hanoi, Vietnam; 4 Department of Hematology, Laboratory Center, 108 Military Center Hospital, Hai Ba Trung, Hanoi, Vietnam; 5 Vietnamese-German Center for Medical Research, 108 Military Center Hospital, Hai Ba Trung Hanoi, Vietnam; King Faisal Specialist Hospital and Research Center, SAUDI ARABIA

## Abstract

**Purpose:**

To establish a highly sensitive and specific approach for the detection of circulating *PIK3CA* H1047R mutation in breast cancer (BC) patients and to investigate the association between the prevalence of *PIK3CA* H1047R mutation and clinical presentations.

**Methods:**

A proper blocker was designed in an allele-specific manner and optimized for PCR*-*based identification of the *PIK3CA* H1047R mutation. The established technique was validated in cell-free DNA samples from 196 recruited BC patients.

**Results:**

The allele-specific PCR assay with a properly designed blocker was able to detect the H1047R mutant variant with 0.01%. By applying the newly established assay, 62 cases (31.6% of the total recruited cases) were found to carry a blood-circulating H1047R mutant. Wherein, the detected mutant rates increased with disease stages from 2/18 (11.1%) of stage I to 17/71 (23.9%) of stage II, 20/53 (37.7%) of stage III, and 23/31 (42.6%) of stage IV (p = 0.025), respectively. Higher frequencies of H1047R mutation were associated with late-stage (p = 0.033) or recurrence (p = 0.045) or metastatic patients (p = 0.049) as well as radiation-treated human epidermal growth factor receptor 2 (HER2) positive BC (p = 0.004). *PIK3CA* mutant carriers were frequently observed in patients under the age of 50 who had liver-metastasized or brain metastases or lymph node-invaded (p < 0.05).

**Conclusion:**

A novel allele-specific PCR assay with high sensitivity was established successfully for the detection of the *PIK3CA* H1047R mutation in clinical practice.

## Introduction

Breast cancer (BC) is the most women-specific malignant disease, in which 70% of the disease is hormone receptor positive (HR-positive) tumours are indicated endocrine-based therapies as standard treatment [1]. However, about 20% of patients acquire resistance to their initial endocrine based regimen, leading to recurrence, metastasis, or even mortality [2]. The disease is specified by stimulation of the PI3K/Akt/mTOR signalling [3–8]. Previous studies have demonstrated that genetic lesions of the *PIK3CA* gene drive such consecutive activation of the PI3K/Akt/mTOR pathway that not only sustain the in the tumor cells’ survival, proliferation and metastasis but also trigger the breast cancer cells to become resistant to the conventional endocrine therapy [9]. Therefore, blocking the PI3K/Akt/mTOR pathway in *PIK3CA*-mutated BC would logically sensitize the *PIK3CA*-mutation carriers to the conventional endocrine regimen [10]. Alpelisib, a PI3K inhibitor, has been approved for the treatment of HR-positive/HER2-negative, *PIK3CA*-mutated, metastatic BC patients [11]. Screening for *PIK3CA* mutations is mandatorily recommended when considering patient candidates for PI3K inhibitor-targeted therapy, especially for cancers with endocrine resistance [12]. *PIK3CA* mutations occur frequently in exons 9 and 20, notably at positions H1047R, E545K, and E542K, which account for 70–80% of *PIK3CA* mutated cases in breast cancer [13]. So, most effort reported so far was to optimize diagnostic assays to identify the mentioned hotspots: H1047R, E545K, and E542K [14–22].

Tumour tissues are traditionally the main sources for DNA extraction and downstream genetic analysis. However, if patients carry tiny tumour masses or relapse with distant metastases, the repeat of tumour biopsy would be a challenge, and the information encoded in tumour tissues is a snap-shot of a given pathological stage, thus it is not suitable for determining the clinical kinetics during patients’ treatments. In these cases, such body fluids as peripheral blood would be an alternative source for DNA analysis; additionally, the analysis of cell-free DNA (cfDNA) from the mentioned biopsy can also be used to monitor the disease progression, tumour burden loads, or patients’ responses to the indicated therapy at a real-time level [23]. One challenge that thwarts the use of liquid biopsy for the identification of clinically relevant genetic lesions is that the amount of extractable cfDNA and the ratio of detectable mutation load are pretty low, even lower than 0.1% of total extractable cfDNA from patients’ plasma [24, 25].

The Sanger sequencing technique can hardly detect mutations with allelic variants lower than 10%; hence, it is rarely used in searching for mutations in plasma samples [26]. Whereas next-generation sequencing (NGS) and digital PCR are examples of current advanced technologies that can be customized to detect a specific panel of somatic mutations, such as the *PIK3CA* gene, with an extremely deep sensitivity [22, 24], the accompanying consumables and equipment cost are high and are unlikely to be widely utilized for routine diagnostics in low-income communities [23].

Asymmetric PCR is an analogue of conventional PCR-based methods wherein the amplification of undesirable or wild-type alleles is blocked by the presence of molecular peptide clamps or oligonucleotide blockers [27]. This art of techniques was optimized for targeted amplification and synchronous detection of either point mutations for instant *KRAS*, *BRAF*, or *JAK2* genes or even mutational hotspots, provided that the mutation hotspots are focused in narrow domains similar to an instant *EGFR* gene [27–30]. In the case of *PIK3CA*, the appearance of the gene mutations in a narrow frame at codons 542, 545, and 1047 allows to utilise asymmetric PCR for detecting these alterations. However, the recently published asymmetric PCR assays hardly acquired a technical sensitivity of about 0.1% (Table 1), which is inadequate to identify the H1047R point mutation in real clinical settings, especially to detect *PIK3CA* mutations in blood.

**Table 1:** Sensitivity of various asymmetric PCR approaches for detecting the *PIK3CA* H1047R mutation.

Methods	Sensitivity (%)	Sample types	Tumour types	Mutation frequency (%)	Authors (Year)
An amplification refractory mutation system (ARMS) PCR	0.1%	Tissue	Breast cancer	15/49 (30.6%)	Ruth E. Board et al. (2008) [17]
Lock Nucleic Acid (LNA) PCR Sequencing	1.3%	Tissue	Breast cancer	30/60 (50%)	Daphne Ang et al. (2012) [21]
An amplification refractory mutation system (ARMS-PCR) using allele-specific scorpion primers	0.5%	Tissue	Breast cancer	15/102 (14.7%)	Alexandre Harlé et al. (2013) [20]
The combination of allele-specific, melting analysis and asymmetric rapid PCR	0.05%	Plasma	Breast cancer	14/76 (18.4%)	Athina Markou et al. (2014) [15]
Peptide Nucleic Acid (PNA) PCR Sequencing	0.2%	Plasma	Colorectal carcinoma	29/128 (22.65%)	Qian Zeng et al. (2017) [31]
Allele-specific competitive blocker-PCR	5%	Tissue	Breast cancer	2/22 (9.1%)	Virginia Alvarez-Garcia et al. (2018) [16]
The nuclease-assisted minor-allele enrichment PCR assay with overlapping probes	0.3%	Plasma	Breast cancer	4/25 (16%)	Leva Keraite et al. (2020) [18]
LNA-modified hairpin-shaped primers	0.1%	Plasma	Colorectal carcinoma	1/2 (50%)	Junsoo Park et al. (2022) [32]

In this study, we proposed an optimized blocker-mediated asymmetric PCR assay integrated with an allele-specific (AS) primer to almost completely inhibit the amplification of a wild-type allele while specifically enriching the signal of a mutant target to get such an ultra-sensitive detection limit of 0.01%. Therefore, our newly established method can be exploited for the identification of the H1047R point mutation from BC patients’ peripheral blood samples.

## Materials and methods

### Clinical samples, sample preparation and DNA extraction

196 over-18-year-old female breast cancer patients (stages I-IV) were recruited from 108 Military Central Hospital (MCH) between June 2021 and June 2023 for this study. Right after hospitalization, written consents to the study were given to individual patients; blood samples, clinical and paraclinical parameters were also collected.

Among the studied cohort, 43 cases (21.9%) suffered recurrence, 54 out of 196 (27.6%) patients were classified as stage IV, and 116 out of 196 (59.2%) patients bear at least one invasive location, either lymph nodes and/or metatases to other organs, as summarized in Table 4 (the patients’ characteristics).

EDTA K_2_-processed peripheral blood samples were centrifuged at 2,000 g for 10 minutes at room temperature, then the separated plasma fractions were collected and frozen until DNA was further used. 500 μL aliquots of plasma were input for individual cfDNA preparation using the MagMAX™ Cell-Free DNA Isolation Kit (Thermo Fisher Scientific, USA). The isolated cfDNA samples were stored at -80°C until further utilization.

The human T-47D breast cancer cell line was purchased from Thermo Fisher Scientific Inc. T-47D cells were maintained in RPMI 1640 medium (Invitrogen, Carlsbad, CA) with phenol red and supplemented with 7.5% fetal bovine serum (FBS) plus 100 units/ml penicillin-streptomycin (Sigma-Aldrich). Cells were cultured and grown in an air-carbon dioxide (95:5) atmosphere at 37°C. Genomic DNA was extracted from the T-47D cells and healthy donors’ white blood cells using the genomic DNA purification kit (Thermo Fisher Scientific) following the manufacturer’s protocol in an elution volume of 100 μl. The extracted DNA was aliquoted and stored at -20°C until use.

A given T-47D cell line number (that contains 50% *PIK3CA* mutant allele [18]) was mixed with white blood cells of healthy donors to formulate a so-called positive cell line dilution series that bears 10%, 1%, 0.1%, 0.01%, 0.001%, and 0% of T-47D cells. These positive cell line dilution series were then input for total genomic DNA extraction. The genomic DNA extracted from the positive dilution series was later used as positive and negative controls for further assay optimization.

### Ethical considerations

The study and its accompanying methods of consent were submitted for regulatory approval to the Institutional Review Board of the 108 Military Central Hospital in Hanoi, Vietnam, and were approved. The Ethical Committee of the 108 Military Central Hospital, Hanoi, provided ethical approval for the study (No. 2527/CN-HDDD). Informed written consent was obtained from all study participants or from their parents or guardians if the study participant was in an unconscious condition. The patients were completely anonymous.

### Primers and oligonucleotides

Primers were designed to amplify amplicons of 86 bp flanking around the studied H1047R mutant, whereas blocker was selected to bone-fine complementarily clamp and inhibit the amplification of wild-type allele (detailed sequences of primers and blocker are listed in Table 2). All oligonucleotides were obtained from the IDT Company (USA). Commercial master mix, nuclease-free water, 6 x loading buffer, and dNTPs were purchased from Thermo Fisher Scientific Inc (USA).

**Table 2:** Oligonucleotides for the real-time PCR assay.

Primer name	Sequence (5’-3’)	Tm (°C)	Final concentration
PIK3CA H1047R mt F	ACAAATGAATGATGCACG	58.5	40 nM
PIK3CA H1047R R	CAGTTCAATGCATGCTGTTTAATT	64.1	40 nM
PIK3CA H1047R wt BL	TGATGCACATCATGG TG/PO4	59	1.2 μM

mt F: mutant specific forward primer; R: common reverse primer; wt BL: wild-type specific blocker.

### Amplification of the *PIK3CA* H1047R point mutation

Allele-specific amplification targeting the *PIK3CA* H1047R point mutation was performed using a real-time PCR system (LightCycler 96, Roche, Switzerland). Primers, blocker, and 2 μl DNA sample were mixed with 2X Universal PCR Master Mix (no UNG) TM (Applied Biosystems, Foster City, CA) in a reaction volume of up to 10 μl. Standard real-time PCR assay thermocycling conditions were used: 10 min. at 95°C, 50 cycles of 15 sec. at 95°C, 20 sec. at 55°C, and 20 sec. at 72°C.

### Statistical analysis

Statistical analysis was performed via SPSS version 20.0 (IBM SPSS Statistics, Armonk, NY, USA). The χ2 and Fisher’s tests were used to determine associations between *PIK3CA* gene mutation and clinicopathological features of BC patients. A p-value ≤ 0.05 was considered to be significant. Graphics were generated with MS Excel 2010 (Microsoft Corporation, Seattle, WA, USA).

## Results

### Assay optimization

**Fig 1:**
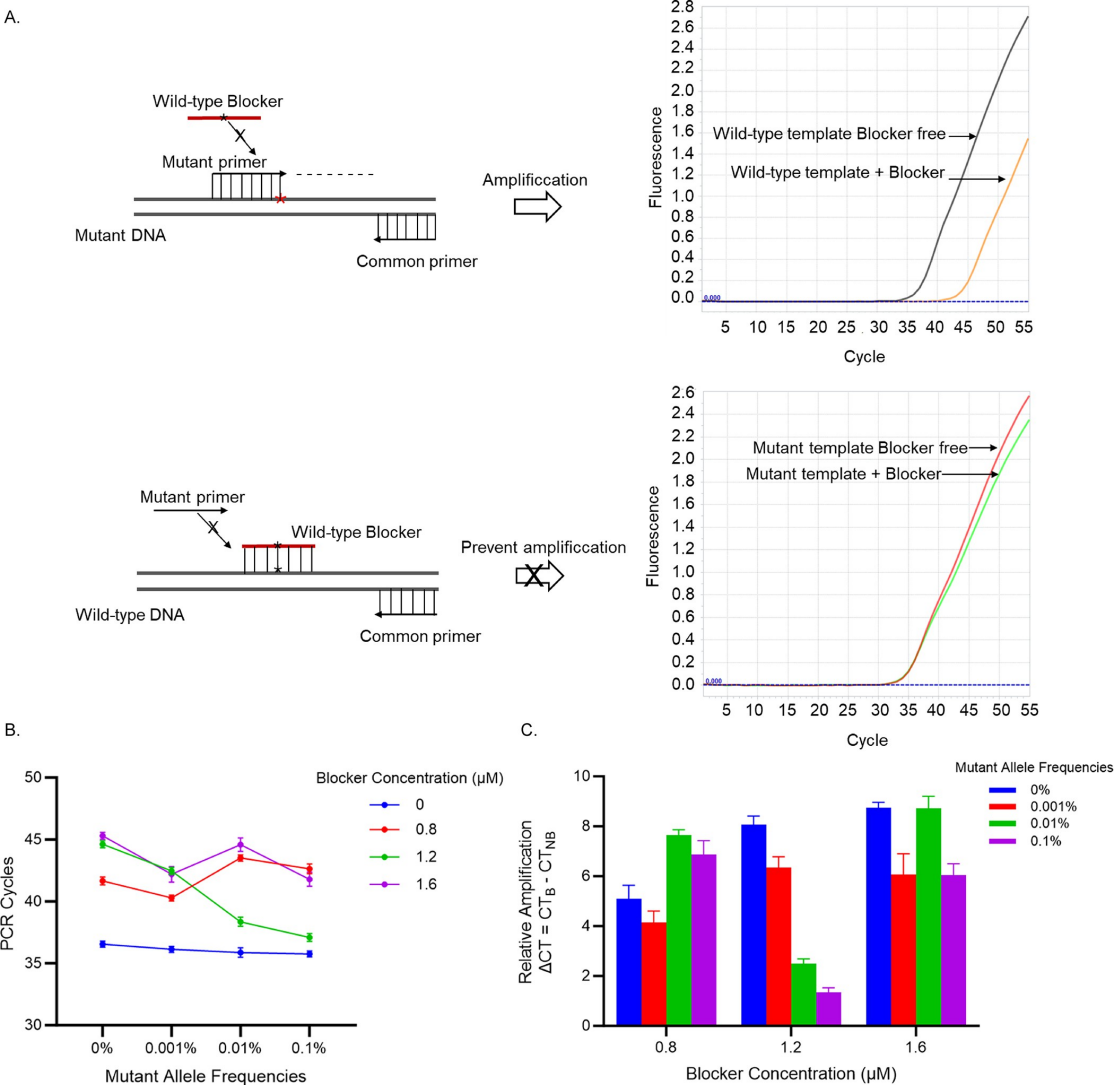
Blocker effect on the polymerase-mediated amplification of targeted amplicons. Upper panel (A): The blocker oligomer was designed to perfectly match the wild-type allele, while the forward primer partially overlapped the blocker binding site, and its 3’ end exactly matched the H1047R mutation site. During the PCR reaction, a perfect blocker/wild-type allele hybrid thwarts the forward primer from annealing to its target, hence suppressing the amplification of the wild-type sequence. On the other hand, the designed blocker bears a mismatched nucleotide to the mutant allele; therefore, a blocker/mutant allele hybrid is weakly formed and hardly prevents the mutant allele’s amplification. Lower panel (B, C): Various blocker concentrations were tested to evaluate the clamping effect on the amplification of wild-type versus mutant alleles. Real-time PCR reactions were performed in triplicate at given blocker concentrations (0, 0.8μM, 1.2μM, 1.6μM); cycle threshold–Ct values (Fig B) were recorded, and ΔCt values (Fig C) were computed as the difference in Ct of the analogous target assays with and without blocker (CT_B_, CT_NB_), respectively. The 1.2μM blocker was selected as the best-optimized parameter for further downstream analysis.

The blocker-mediated PCR clamping system is schematically presented in Fig 1A. A forward primer (mutant-specific primer) is perfectly matched to the mutant allele, while a 3′ phosphorylated, un-extended oligonucleotide sequence (wild-type blocker) that is perfectly complementary to the wild-type sequence on the same strand was used as the blocker to inhibit the polymerase-mediated amplification of the wild-type allele. Various blocker concentrations (0, 0.8 μM, 1.2 μM, 1.6 μM) were tested to evaluate the clamping effect on the amplification of wild-type versus mutant alleles. At a concentration of 1.2 μM, the blocker acquires its sharpest inhibitory effect on the amplification of the wild-type allele while keeping the mutant allele almost intact. Hence, 1.2 μM of the blocker was selected as the optimized parameter for further downstream analysis (Fig 1B and 1C).

### Detection limit optimization for identifying the *PIK3CA* H1047R mutation

**Fig 2:**
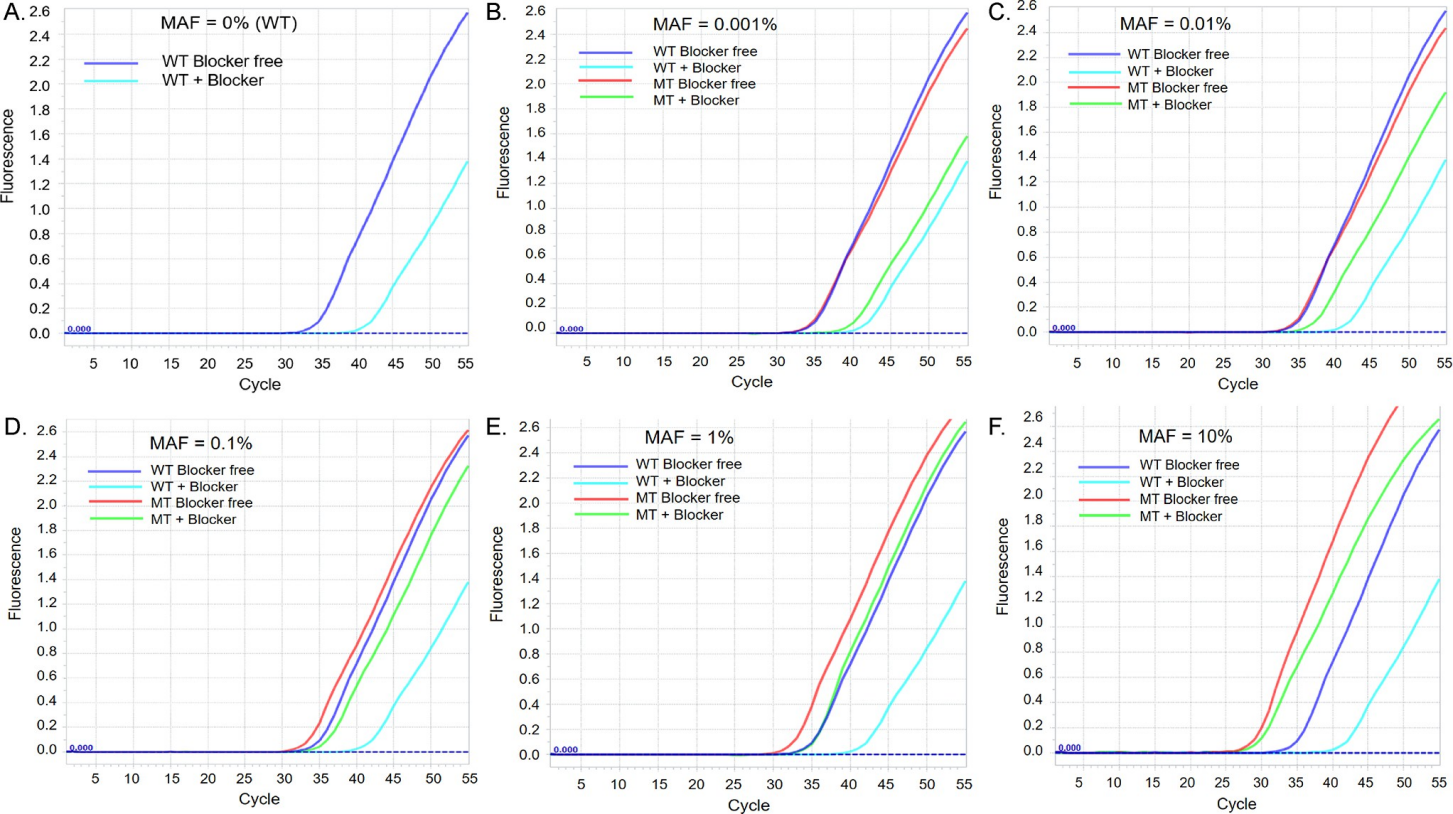
Real-time PCR assays to optimize the LOD for identifying the *PIK3CA* H1047R mutation. The total genomic DNA extracted from T-47D cell positive cell line dilution series that bear mutant allele frequency (MAF) of 10%, 1%, 0.1%, 0.01%, 0.001%, and 0% were used as input templates for corresponding real-time PCR assays. At each dilution point, the real-time PCR was performed in four conditions: (i) wild-type template without blocker (blocker-free); (ii) wild-type template with blocker; (iii) mutant template (0.001%, 0.01%, 0,1%, 1%, 10%) without blocker; and (iv) mutant template (0.001%, 0.01%, 0,1%, 1%, 10%) with blocker.

The total genomic DNA extracted from T-47D positive cell line (known to carry 50% H1047R [18]) was mixed with an equal amount of total genomic DNA extracted from healthy donors’ white blood cells to form dilution series that bear 10%, 1%, 0.1%, 0.01%, 0.001%, and 0% of H1047R. These dilution series were used as input templates for validating the technical sensitivity and the specificity of the designed real-time PCR assay. At each dilution point, the real-time PCR was performed in four conditions: (i) wild-type template without blocker (blocker-free); (ii) wild-type template with blocker; (iii) mutant template (0.001%, 0.01%, 0,1%, 1%, 10%) without blocker; and (iv) mutant template (0.001%, 0.01%, 0,1%, 1%, 10%) with blocker, and was repeated 15 times to determine the assay’s technical sensitivity (the limit of detection, LOD). At the lowest concentration of the mutant allele (0.001%), the acquired ΔCt value distance is 5.19 (p = 0.714), and the ΔCt distance linearly decreases to 1.22 (p = 0.001) at the highest dilution point of the 10% mutant allele (Fig 2 and Table 3). Because only assays with a p-value lower than 0.05 are considered statistically significant [33], the 0.01% mutant allele is concluded to be the LOD of our newly established technique, and the assay’s accuracy was maintained at 0.01% or higher H1047R mutation.

**Table 3:** Assay reproducibility and LOD.

Repeated optimal assays (n = 15)	Mutant allele frequency (%)					
	0% (WT)	0.001%	0.01%	0.1%	1%	10%
Ct (no blocker)	39.71	38.19	37.68	37.00	35.10	30.76
Ct (blocker)	45.28	43.38	41.77	38.83	36.51	31.98
Δ Ct	5.57	5.19	4.09	1.83	1.41	1.22
p-value		0.714	0.041*	0.003*	0.003*	0.001*

*Statistically significant value, p < 0.05

p-value: mutant H1047R versus wild-type H1047R

Real-time PCR assays with or without blocker were performed fifteen times on dilution series of 10%, 1%, 0.1%, 0.01%, 0.001%, and 0% mutant. ΔCt values were computed as the difference in Ct of the analogous target assays with and without blocker.

### *PIK3CA* mutation determined in BC patients’ plasma samples

By applying the newly established assay to 196 institutionally recruited BC patients’ plasma samples, 62 cases (31.6% of total recruited cases) harboured the H1047R mutant (Table 4); the detected mutant rates increased with disease stages from 2/18 (11.1%) of stage I to 17/71 (23.9%) of stage II, 20/53 (37.7%) of stage III, and 23/31 (42.6%) of stage IV (p = 0.025), respectively (Table 4 and S1A Fig). However, there was no relationship between the H1047R mutation and the patient’s age, family history, menopausal status, HR or HER2 expressions, or tumour histopathology (Table 4). On the other hand, patients with recurrence, metastasis, visceral metastasis, bone metastases, and patients with multiple metastatic sites have a higher incidence of *PIK3CA* gene mutations (S1B and S1C Fig). Our data also revealed that individuals with late-stage cancer (p = 0.033), recurrence (p = 0.045), metastasis (p = 0.049), or liver metastatic disease (p = 0.034) or brain metastases (p = 0.009), as well as radiation-treated HER2 positive BC (p = 0.004) were more likely to have the H1047R mutation (Table 4, S1 and S2B Figs); HR-positive/HER2-negative advanced BC patients who were treated selective estrogen receptor modulators (SERMs, 45.5%) or selective estrogen receptor degraders (SERDs, 36.4%) had a higher percentage of the *PIK3CA* H1047R mutation than those who were free of SERMs or SERDs treatment (36.4% and 13.6%, respectively), and no AI-recipients carried the mutation (S2A Fig). There is no significant association found between SERMs/SERDs and the *PIK3CA* H1047R mutation (p > 0.05) (S2A Fig). Especially, *PIK3CA* mutant carriers were strongly associated with patients under the age of 50 who had liver-metastasized or brain metastases or lymph node-invaded (p < 0.05) (S3 Fig).

**Table 4:** Characteristics of the study population according to circulating *PIK3CA* H1047R mutation status.

Variable	Total n = 196 (100%)	H1047R		p-value^#^
		Mutant n = 62 (31.6%)	Wild-type n = 134 (68.4%)	
**Age at diagnosis**	52.43 ± 12.38	50.32±11.97	53.41±12.49	0.105
**Family history**				
Yes	38 (19.4)	11 (17.7)	27 (20.1)	0.846
No	158 (80.6)	51 (82.3)	107 (79.9)	
**Disease stages**				
I	18 (9.2)	2 (3.2)	16 (11.9)	**0.025** *
II	71 (36.2)	17 (27.4)	54 (40.3)	
III	53 (27)	20 (32.3)	33 (24.6)	
IV	54 (27.6)	23 (37.1)	31 (23.1)	
**Tumour histology**				
Ductal	191 (97.4)	62 (100)	129 (96.3)	0.181
Lobular	5 (2.6)	0 (0)	5 (3.7)	
**Grade**				
1	12 (6.1)	3 (6.1)	9 (6.7)	0.797
2	108 (55.1)	36 (58.1)	72 (53.7)	
3	76 (38.8)	23 (37.1)	53 (39.6)	
**Menopausal status**				
Post-menopausal	109 (55.6)	31 (50)	78 (58.2)	0.354
**HR status**				
Positive	148 (75.5)	50 (80.6)	98 (73.1)	0.255
Negative	48 (24.5)	12 (19.4)	36 (26.9)	
**HER2 status**				
Positive	120 (61.2)	42 (67.7)	78 (58.2)	0.212
Negative	76 (38.8)	20 (32.3)	56 (41.8)	
**Recurrence**				
Yes	43 (21.9)	19 (30.6)	24 (17.9)	**0.045** *
No	153 (78.1)	43 (69.4)	110 (82.1)	
**Metastatic disease**				
Yes	116 (59.2)	43 (69.4)	73 (54.5)	**0.049** *
No	80 (40.8)	19 (30.6)	61 (45.5)	
**The number of metastasis lesion**				
0	80 (40.8)	19 (30.6)	61 (45.5)	0.131
≤ 2	94 (48)	34 (54.8)	60 (44.8)	
≥ 3	22 (11.2)	9 (14.5)	13 (9.7)	
**Metastatic sites**				
Lymph nodes	104 (53.1)	38 (61.3)	66 (49.3)	0.126
Lung	26 (13.3)	9 (14.5)	17 (12.7)	0.821
Liver	7 (3.6)	5 (8.1)	2 (1.5)	**0.034** * ^**a**^
Brain	4 (2.0)	4 (6.5)	0 (0)	**0.009** * ^**a**^
Bone	29 (14.8)	12 (19.4)	17 (12.7)	0.279
**Treatment**				
Surgical therapy	161 (82.1)	49 (79)	112 (83.6)	0.431
Hormone therapy	98 (50)	32 (51.6)	66 (49.3)	0.878
Chemotherapy	175 (89.3)	57 (91.9)	118 (88.1)	0.469
Radiotherapy	60 (30.6)	25 (40.3)	35 (26.1)	**0.045** *

*Statistically significant value, p < 0.05

p-value^#^: mutant versus wild-type

a: Fisher’s Exact Test

HER2: human epidermal growth factor receptor-2; HR: hormone receptor.

## Discussion

Clinical samples are normally heterogeneous in terms of having both tumour and normal cells, or both wild-type and mutant DNA alleles. The mutation load may occasionally be below the detection threshold of diagnostic tools when the disease is at an early stage. Therefore, it is necessary to establish high-sensitivity diagnostic tools suitable for deployment in routine diagnostic conditions, especially for patient samples with low DNA quantities obtained from plasma.

Asymmetric PCR is one of the methods available for identifying gene mutations, including allele-specific priming in combination with competitive oligonucleotides to block the amplification of wild-type alleles that have been deployed for the detection of various mutation targets [27–29]. However, in some cases, the blocking of wild-type alleles is not specific enough to generate a significant signal amplification difference between the targets and unwanted alleles. In the case of the H1047R mutation model, most of the previously reported asymmetric PCR assays hardly acquired enough sensitivity to be robustly implemented in routine clinical diagnostics, especially to detect circulating H1047R in patients’ blood samples. We tactfully designed an AS-PCR assay with an optimized blocker to weakly inhibit the PCR signal of the mutant target while strongly clamping amplification of wild-type sequences, hence leading to the acquisition of a 0.01% mutant allele detection limit (LOD) in a DNA cell line model. Most earlier studies did not reveal any methods that were superior to the novel technique mentioned here (Table 1).

With the use of the newly established assay, we identified 31.6% (62/196) of recruited BC’s plasma carrying the *PIK3CA* H1047R mutation. This mutation rate is in accordance with previous findings from other ethnicities [13, 17, 34]. In our study cohort, the *PIK3CA* mutation was related to disease progression or worse illness in patients, which is consistent with prior research [18, 25–37]. Metastatic BC patients with *PIK3CA*-mutated HR-positive/HER2-negative tumours exhibit a poor prognosis and hormone resistance [36, 38, 39]. Our data also revealed that the H1047R mutation was considerably more common in SERMs/SERDs-received HR-positive /HER2-negative advanced BC patients or in radiotherapy-treated HER2 positive individuals, which strengthens the potential utility of cfDNA *PIK3CA* mutations as a tumour marker to guide treatment selection.

On the other hand, the current study embeds a number of limitations, such as a cross-sectional design with a limited patient size, being a single-institution study, and no matching-tumor analysis. Additionally, we only tested for the *PIK3CA* H1047R hotspot mutation, and the method does not provide a quantitative evaluation of initial mutant allele frequency, which is important for quantitative monitoring of patients’ responses during a given treatment.

## Conclusion

A novel allele-specific PCR assay was established for the detection of the *PIK3CA* H1047R mutation from the patients’ plasma in the clinical setting. The H1047R mutation is more common in advanced Vietnamese BC patients, especially those with liver invasion or brain metastases, or HER2-positive BC who are treated with radiation.

## Supporting information

S1 Fig Clinical data on the detected *PIK3CA* H1047R mutation. Prevalence of the circulating *PIK3CA* H1047R mutation in different stages of breast cancer (A), in groups based on recurrence and metastatic status (B), and the number of metastatic lesions (C). * p < 0.05. RE: recurrence; MS: metastatic disease; VS: visceral metastasis; Liver: liver metastasis; Lung: lung metastasis; Brain: brain metastasis; LN: lymph node invasion; Bone: bone metastasis.
(TIF)

S2 Fig Distribution of the circulating *PIK3CA* H1047R mutation in subgroups with different therapies. Prevalence of the circulating *PIK3CA* H1047R mutation in HR-positive/HER2-negative advances breast cancer (HR+/HER2- ABC) with different endocrine regimens (A) and in HER2-positive breast cancer (HER2+ BC) with different treatments (B). SERMs: selective estrogen receptor modulators; SERDs: selective estrogen receptor degraders; AI: Aromatase inhibitors. * p < 0.05.
(TIF)

S3 Fig Distribution of the circulating H1047R mutation by age groups. The *PIK3CA* H1047R mutant frequency in groups ≤ 50 years old (A) and > 50 years old (B), respectively. RC: recurrence; MS: metastatic disease; LN: lymph node invasion; Liver: liver metastasis; Lung: lung metastasis; Brain: brain metastasis; Bone: bone metastasis. * p < 0.05.
(TIF)
