# Supplementary figures and images for: Identification of breast cancer-associated PIK3CA H1047R mutation in blood circulation using an asymmetric PCR assay
Source: PLoS One. 2024 Aug 28;19(8):e0309209. doi: 10.1371/journal.pone.0309209 (PMC11356436; doi:10.1371/journal.pone.0309209)

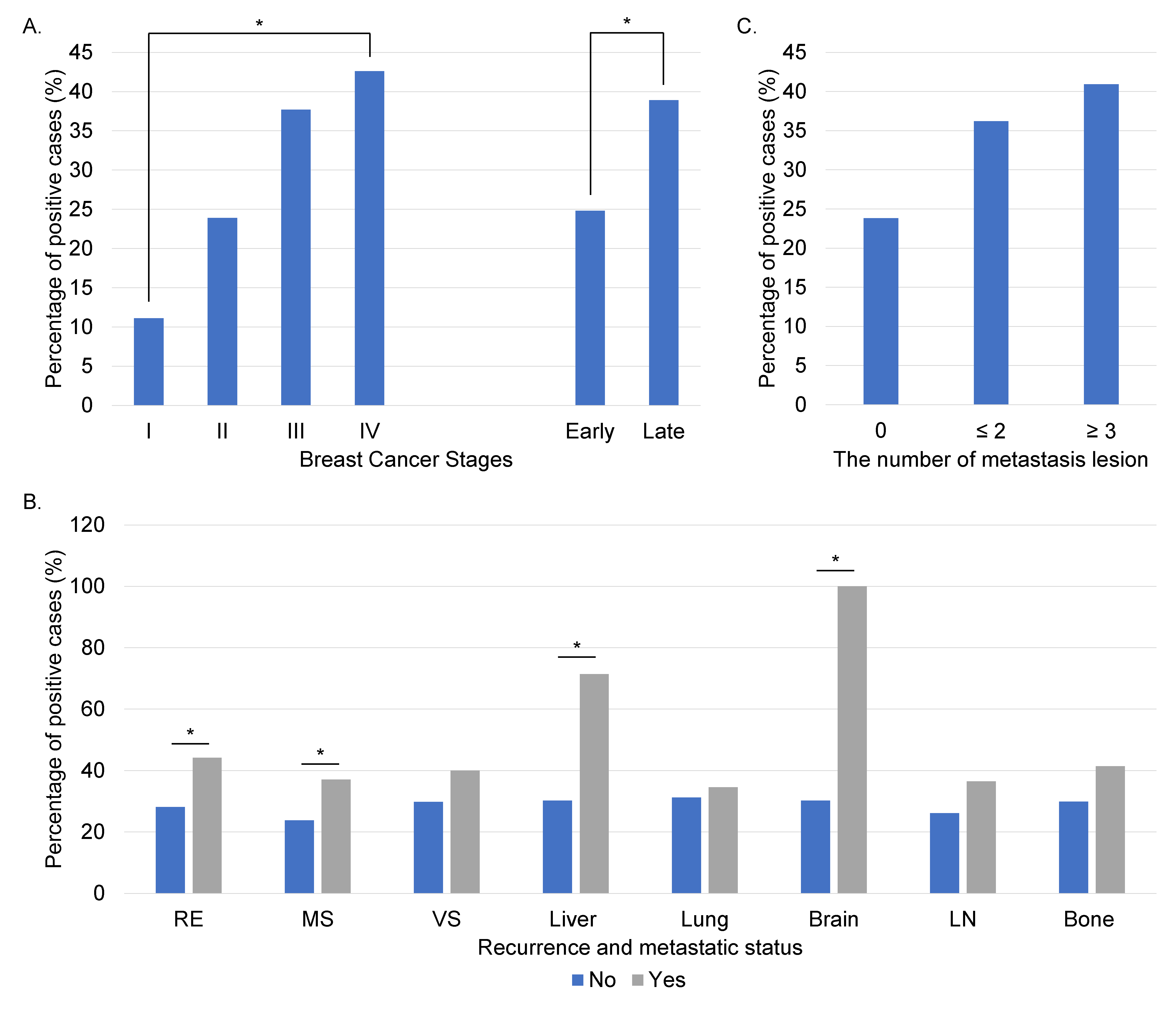

Supplement: S1 Fig — Prevalence of the circulating PIK3CA H1047R mutation in different stages of breast cancer (A), in groups based on recurrence and metastatic status (B), and the number of metastatic lesions (C). * p < 0.05. RE: recurrence; MS: metastatic disease; VS: visceral metastasis; Liver: liver metastasis; Lung: lung metastasis; Brain: brain metastasis; LN: lymph node invasion; Bone: bone metastasis. (TIF) [file pone.0309209.s001.tif]

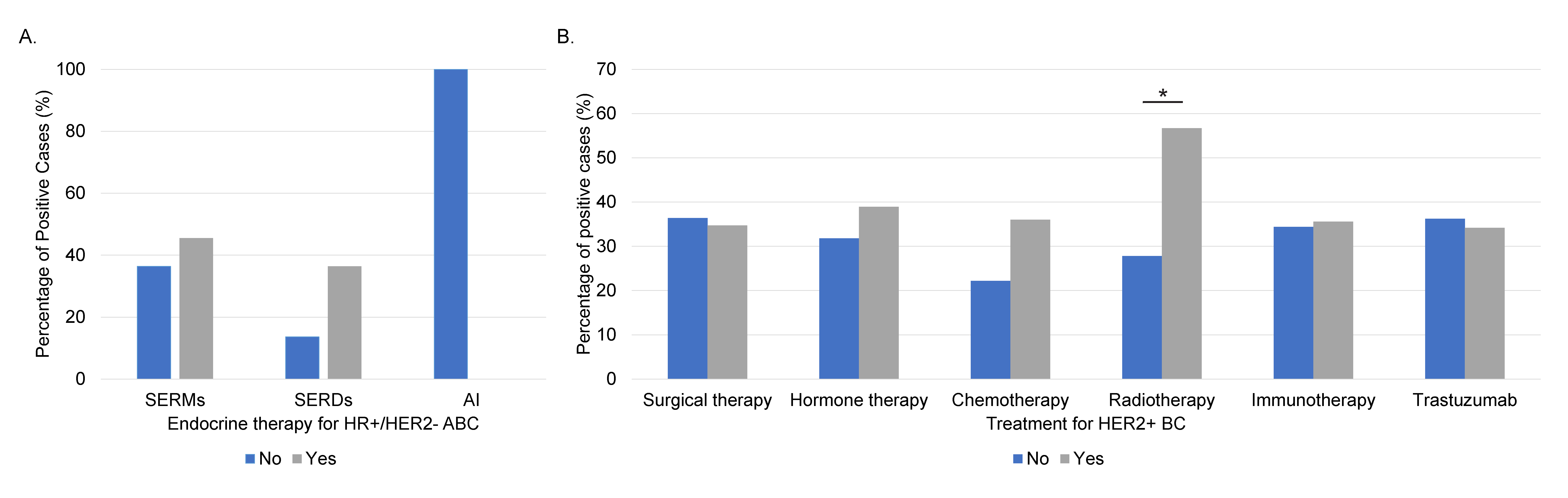

Supplement: S2 Fig — Prevalence of the circulating PIK3CA H1047R mutation in HR-positive/HER2-negative advances breast cancer (HR+/HER2- ABC) with different endocrine regimens (A) and in HER2-positive breast cancer (HER2+ BC) with different treatments (B). SERMs: selective estrogen receptor modulators; SERDs: selective estrogen receptor degraders; AI: Aromatase inhibitors. * p < 0.05. (TIF) [file pone.0309209.s002.tif]

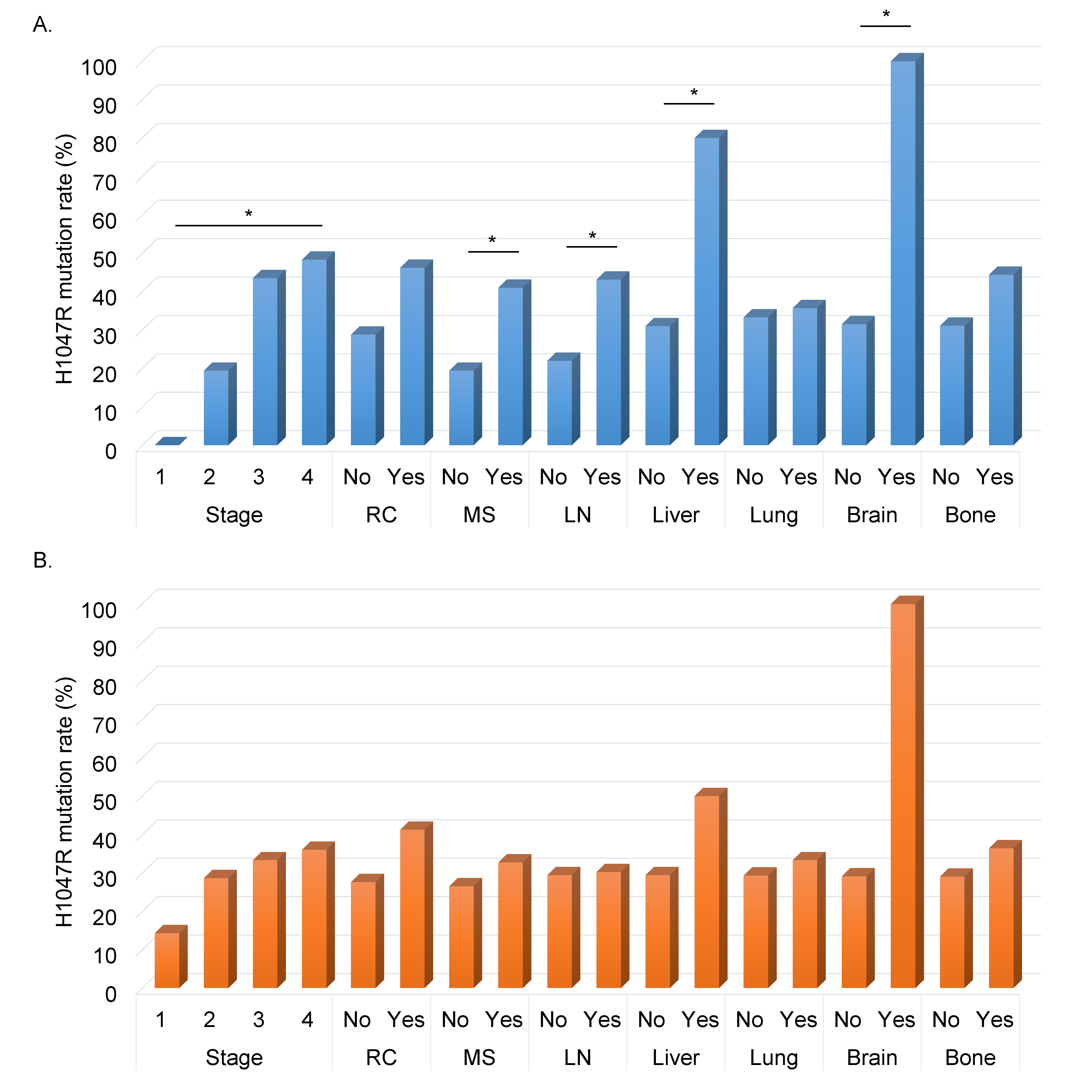

Supplement: S3 Fig — The PIK3CA H1047R mutant frequency in groups ≤ 50 years old (A) and > 50 years old (B), respectively. RC: recurrence; MS: metastatic disease; LN: lymph node invasion; Liver: liver metastasis; Lung: lung metastasis; Brain: brain metastasis; Bone: bone metastasis. * p < 0.05. (TIF) [file pone.0309209.s003.tif]
